# Pooled Analysis of Real-World Evidence Supports Anti-CGRP mAbs and OnabotulinumtoxinA Combined Trial in Chronic Migraine

**DOI:** 10.3390/toxins14080529

**Published:** 2022-08-01

**Authors:** Damiana Scuteri, Paolo Tonin, Pierluigi Nicotera, Marilù Vulnera, Giuseppina Cristina Altieri, Assunta Tarsitano, Giacinto Bagetta, Maria Tiziana Corasaniti

**Affiliations:** 1 Pharmacotechnology Documentation and Transfer Unit, Preclinical and Translational Pharmacology, Department of Pharmacy, Health and Nutritional Sciences, University of Calabria, 87036 Rende, Italy; 2 Regional Center for Serious Brain Injuries, S. Anna Institute, 88900 Crotone, Italy; patonin18@gmail.com; 3 German Center for Neurodegenerative Diseases (DZNE), 53127 Bonn, Germany; pierluigi.nicotera@dzne.de; 4 Pharmaceutical Service, Provincial Health Authority (ASP), 87100 Cosenza, Italy; marilu.vulnera@tiscali.it (M.V.); gcaltieri@gmail.com (G.C.A.); 5 Pain Therapy Center, Provincial Health Authority (ASP), 87100 Cosenza, Italy; assunta.tarsitano@aspcs.it; 6 Department of Health Sciences, University “Magna Graecia”, 88100 Catanzaro, Italy; mtcorasa@unicz.it

**Keywords:** onabotulinumtoxinA, migraine, anti-CGRP monoclonal antibodies, PRISMA 2020, pooled analysis

## Abstract

OnabotulinumtoxinA, targeting the CGRP machinery, has been approved for the last two decades for chronic migraine prevention. The recently approved monoclonal antibodies (mAbs) directed towards the calcitonin gene-related peptide (CGRP) pathway open a new age for chronic migraine control. However, some 40% patients suffering from chronic migraine is still resistant to treatment. The aim of this work is to answer the following PICOS (participants intervention comparator outcome study design) question: Is there evidence of efficacy and safety of the combined administration of anti-CGRP mAbs and onabotulinumtoxinA in chronic migraine? A systematic review and meta-analysis [Preferred Reporting Items for Systematic Reviews and Meta-Analyses (PRISMA) 2020 recommendations] was made up to 19 April 2022. The results are encouraging: the combined treatment proved to afford ≥50% monthly headache days (MHDs)/frequency reduction respect to baseline in up to 58.8% of patients; in comparison, anti-CGRP mAbs reduce MHDs of 1.94 days from baseline and botulinum toxin of 1.86 days. Our study demonstrates for the first time that the combination therapy of onabotulinumtoxinA with anti-CGRP mAbs affords a reduction of 2.67 MHDs with respect to onabotulinumtoxinA alone, with moderate certainty of evidence. Adequately powered, good-quality studies are needed to confirm the response to combination therapy in terms of efficacy and safety. PROSPERO registration: CRD42022313640.

## 1. Introduction

Rationale and Objective

**Figure 1:**
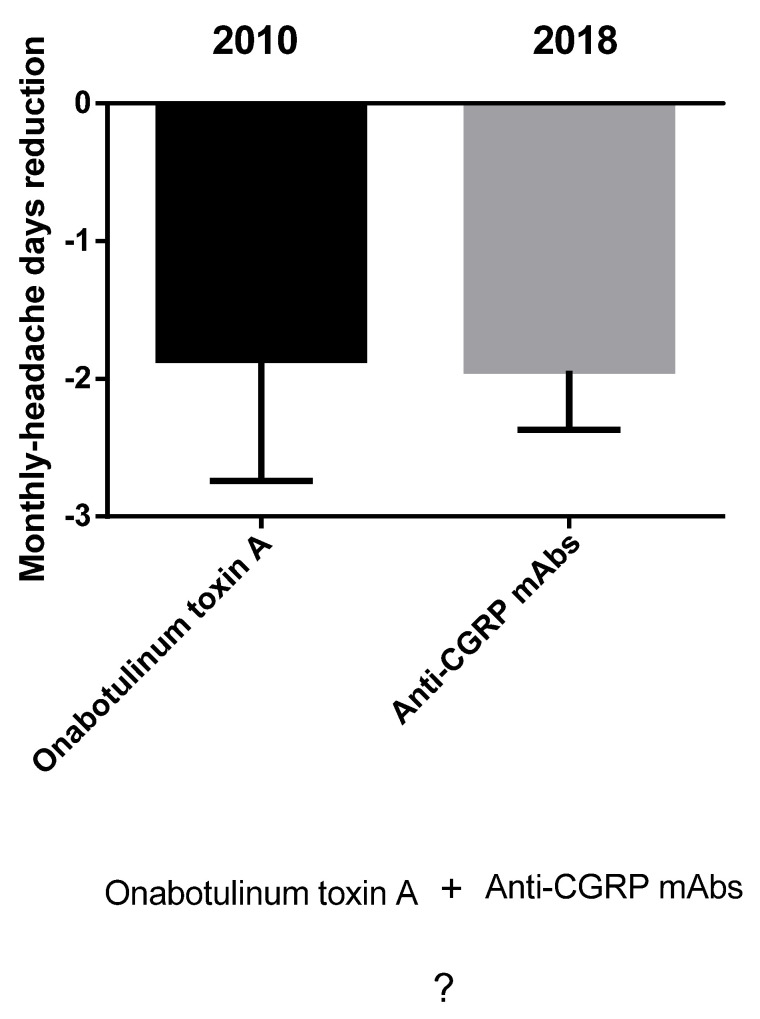
Indirect comparison between onabotulinumtoxinA and antibodies directed towards the signaling of CGRP. Anti-CGRP mAbs reduce the number of monthly headache days from baseline of 1.94 days and botulinum toxin of 1.86 days [36]. How much reduction the combination of the two treatments can afford is to be discovered. Data are expressed as monthly headache days (MHDs) difference over baseline ± upper/lower limit of confidence interval (CI).

Migraine is one of the most frequent and debilitating neurological disorders, accounting for 72% of all neurological disease years lived with disability (YLDs) [1]. The attack lasts from 4 to 72 h and consists of moderate-to-severe unilateral throbbing headache pain accompanied by photophobia, phonophobia, nausea, vomiting, movement sensitivity and allodynia; it is preceded by a prodromic phase with or without aura, characterized by transient focal neurological symptoms, and followed by a postdromic stage [2]. Migraine consists of a spectrum of illnesses along a *continuum* [3,4] of increasing cortical excitability [5], leading to a chronic disease during which episodic manifestations occur (CDEM) [6]. The latter increases in frequency across the life span [7] up to chronic migraine; it is characterized by over 15 days of headache per month, of which there are at least 8 days of migraine, for at least three months [2]. Therefore, patients suffering from frequent attacks need daily prophylactic treatment for chronic migraine to reduce the number and severity of acute episodes and delay attacks, increasing the pain-free and most-bothersome-symptom-free interictal period [8]. The molecular cloning of the calcitonin gene-related peptide (CGRP), belonging to the six-member family of the calcitonin petides including calcitonin, adrenomedullin 1 and 2, amylin and CGRP α and β [9,10], together with the discovery of its role in pain modulation [11], in meningeal vasodilation [12] and in sensitization of the trigeminal ganglion [13] raised interest in the pathway of this neurotransmitter as target for migraine treatment and prevention of chronification. One of the medications interfering with CGRP machinery is the onabotulinumtoxinA, approved by the Food and Drug Administration (FDA) in 2010 for the prophylactic treatment of chronic migraine [14], after the results of the the Phase III Research Evaluating Migraine Prophylaxis Therapy (PREEMPT) I and II (NCT00156910, NCT00168428) studies [15,16,17], and recommended by the UK National Institute for Health and Care Excellence (NICE) for patients not responding to at least three prior preventative treatments. The onabotulinumtoxinA blocks the release of CGRP, cleaving the 25 kDa synaptosomal-associated protein (SNAP-25), needed for the neurotransmitter exocytosis [18]. In particular, the onabotulinumtoxinA can also reduce the need for rescue medications [19,20] through this mechanism and its forms can increase the analgesic efficacy in experimental neuropathic conditions [21]. In fact, botulinum toxin type A reverses mechanical hypersensitivity of sensitized C-units interfering with neuronal surface expression of high-threshold mechanosensitive ion channels linked preferentially to mechanical pain by preventing their fusion into the nerve terminal membrane [22]. In addition, as demonstrated by microdialysis for glutamate in the rat formalin model, the possible antinociceptive action of botulinum toxin type A also lies in its property to inhibit neurotransmitter release from primary sensory neurons [23]. In fact, botulinum toxin is a multipurpose drug able to provide long-lasting relief in several forms of pain, migraine and primary headache [24,25]. More recently, specific monoclonal antibodies (mAbs) directed towards CGRP ligand (fremanezumab, galcanezumab and eptinezumab, the only administered intravenously and with potential for acute onset of action in attacks [26,27,28,29]) or its receptor complex (erenumab) were developed and approved between 2018 and 2020 by the FDA and the European Medicines Agency (EMA) for the preventive treatment of episodic and chronic migraine [30] to be added to ≥1 established preventive treatment [31]. Unfortunately, even if the anti-CGRP mAbs provide pain relief to difficult-to-treat patients [32], some 40% of nonresponders is present [33]. Research is still scarce in the recognition of the mechanisms underlying this resistance, which could be, at least in part, affected by polymorphisms [34]. The possible sinergy of onabotulinumtoxinA and anti-CGRP mAbs in the management of patients resistant to treatment should be investigated in real-world context. Moreover, the prevalence of migraine in Italy standardized per age is the highest calculated, according to the the Global Burden of Disease Study 2016, ranging from 20.000 to 21.000 patients per 100.000 population [35]. A recent indirect comparison study found that the use of anti-CGRP mAbs reduces of 1.94 days the number of monthly headache days (MHDs) from baseline (*p* < 0.00001) and botulinum toxin of 1.86 days (*p* < 0.0001) [36] (Figure 1). How much reduction the combination of the two treatments could afford to increase the responders’ rate is to be established.

To answer this question, the present systematic review and pooled analysis has two purposes: (1) to investigate for the first time the pharmacoepidemiological data of prescriptions of onabotulinumtoxinA and anti-CGRP mAbs in the real-world setting of the Calabria region; (2) to investigate the international real-world evidence of efficacy and safety of the concurrent treatment with onabotulinumtoxinA and anti-CGRP mAbs in the prevention of chronic migraine through systematic search and pooled analysis according to the most recently updated Preferred Reporting Items for Systematic reviews and Meta-Analyses (PRISMA) 2020 recommendations [37], for studies assessing the effect of health interventions independently on their design.

## 2. Results

### 2.1. Real-World Evidence

The results obtained from the pharmaceutic service highlight a reduction in the use of onabotulinumtoxinA in favor of anti-CGRP mAbs over the period of 2020–2022. In fact, 100 100-Unit vials were prescribed to 12 patients within 2020. On the contrary, within 2021, only erenumab, galcanezumab and fremanezumab were prescribed. No prescription of eptinezumab was recorded. In particular, 42 patients were treated in the year, instead of the 12 of the previous year. Among these:

- 11 patients received erenumab 70 mg, for a total of 125 doses, thus accounting for one administration per month for 11–12 months;
- 12 patients were prescribed erenumab 140 mg, for a total of 134 doses, accounting for one administration per month for 11 months: therefore, patients most likely moved to this treatment after one month of lower dosage;
- 11 patients received 131 doses of galcanezumab 120 mg, one per month a year;
- 8 patients were treated with 72 doses of fremanezumab 225 mg and 1 dose of fremanezumab 675 mg, accounting for one year-long treatment.

Data collection ended by March 2022, highlighting a wide increase in patients treated for chronic migraine, since 51 patients received anti-CGRP mAbs presciptions, a lot more patients in 2 months of 2022 than in the whole year 2021. In detail, five patients received 5 vials of erenumab 70 mg, underlying a further switch of many patients to the highest dosage. A total of 20 patients were prescribed 57 doses of erenumab 140 mg, thus one per month, and 19 patients were prescribed a comparable amount of doses of galcanezumab 120 mg. Only seven patients received one dose per month of fremanezumab 225 mg. None of the extracted results suggests a combination therapy of onabotulinumtoxinA and anti-CGRP mAbs in this real-world setting. Based on the sample size of the present health district, consisting of 298,000 inhabitants, over 213,000 are under 60 years of age, hence the segment most affected by migraine, and according to age-standardized prevalence of migraine in Italy, which is the highest calculated in the Global Burden of Disease Study 2016, ranging from 20.000 to 21.000 patients affected per 100.000 population [35], migraine results under diagnosed and under treated in the present real-world context.

### 2.2. Selection of the Studies

**Figure 2:**
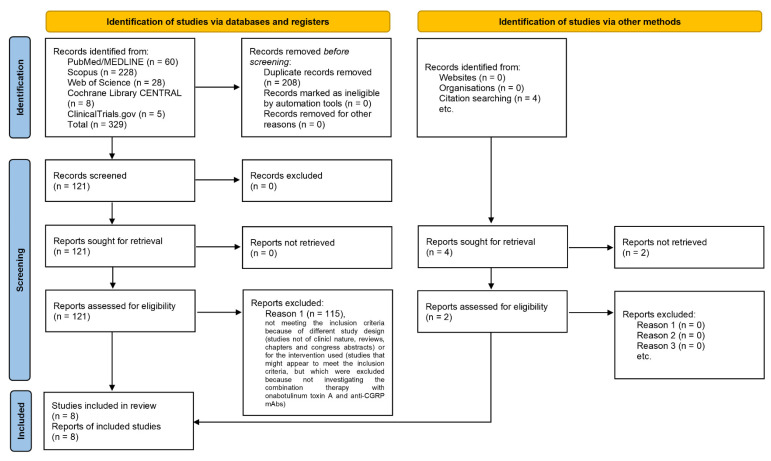
PRISMA 2020 flow diagram [37,42], based on flow diagrams by Boers [43], Mayo-Wilson et al. [44] and Stovold et al. [45], reporting the process of identification and selection of the studies eligible for the systematic review and quantitative analysis.

The database search retrieved 329 total results: 60 records were obtained from PubMed/MEDLINE, 228 from Scopus, 28 from Web of Science, 8 from the Cochrane Library CENTRAL database and 5 from Clinicaltrials.gov. Further screenings, in particular a reference list search, retrieved four more records: (1) the study by Cohen et al., 2021 [38], but its report was not available; (2) the abstract by Singh et al. [39], without complete study; (3) the retrospective observational case series by Ozudogru et al. [40]; (4) the real-world observational study by Boudreau [41]. The 329 records obtained were searched for duplicates. After the removal of duplicates, 121 results were left to screen. The latter were sought, screening title and abstract, leaving six results to assess for eligibility to add to the two results retrieved from reference list screening. In fact, 115 studies were excluded, not meeting the inclusion criteria because of different study design (studies not of clinical nature, reviews, chapters and congress abstracts) or for the intervention used (studies that might appear to meet the inclusion criteria, but were excluded because they did not investigate the combination therapy with onabotulinumtoxinA and anti-CGRP mAbs). Therefore, only eight studies were included in the analysis. The flow diagram illustrating the process of database searching and record screening and selection is reported in Figure 2.

### 2.3. Qualitative Analysis

The eight articles eligible for analysis were grouped and analyzed according to the Cochrane Consumers and Communication Review Group guidelines. A summary of the main characteristics of the studies investigated is reported in Table 1, illustrating: the report (author and year); the study design and sample size; the participants, based on type of migraine and of treatments; the research design with treatment assignment, allocation and concealment mechanisms and length of follow-up; the intervention type, timing and dose; the outcomes, results and authors’ conclusions.

In the study of Armanious et al., 2021 [46], the combination therapy provided a significant mean decrease of 8.1 MHDs (*p* < 0.001) and 30% reduction [7.4 MMDs (*p* < 0.001)] at 90 days. The study by Blumenfeld et al., 2021 [47] reported a primary analysis cohort (*n* = 257) and a sensitivity analysis cohort (*n* = 172), including only patients with at least four MHDs at baseline and at least moderate headache-related disability [Migraine Disability Assessment (MIDAS) score > 11 or 6-item Headache Impact Test (HIT-6) score > 50]; therefore, the primary analysis cohort only was included in the analysis, due to preset eligibility criteria. According to the latter retrospective chart, one-third (31.5–36.7%) of patients presented a ≥50% reduction in MHDs after ~6–12 months, with a ≥5-point reduction from baseline in 43.7–45.1% cases and a ≥30% reduction in migraine-related disability according to MIDAS score for 27.1–29.6% patients. In fact, after ~6–12 months of combination therapy, the mean MIDAS scores were significantly reduced from baseline by 6.1 to 11.1 points. The safety outcome is reported in this study showing that sixty-eight out of two hundred and forty-five (the 27.8% of the total sample) (68/245) patients presented adverse events, with the most common being constipation, most commonly with erenumab. The study of Boudreau 2020 [41] is the only prospective, observational study retrieved (NCT04152434), assessing the primary outcome of the reduction in migraine days’ frequency and the secondary outcome of adverse events’ presentation. Interestingly, the 65% of patients treated with the combination therapy achieved a reduction in migraine frequency, which was a much higher percentage than the 26% obtained with erenumab alone and than the 15% with erenumab in combination with prophylactic treatments other than botulinum toxin A. The study by Mechtler et al. [48] presents the same inclusion criteria of the retrospective chart by Blumenfeld et al., 2021 [47], as well as the same outcomes, outcome measures and time points of 3-6-9 and 12 months of investigation. However, the paper by Mechtler reports that since paired HIT-6 and MIDAS scores from baseline and post-index assessments were only available for up to four patients, no further analyses were reported for those outcome measures. After 12 months of combination therapy, MHD decreased by a mean of 4.6 days and 34.9% patients achieved a ≥50% reduction in MHD. Also in this case, the most common adverse events were constipation and injection-site reactions. The retrospective cohort study by Nandyala et al., 2022 [49] reported a significant reduction in MMDs (11.3 ± 9.3 vs. 14.9 ± 9.4, *p* < 0.001) and of MHDs (18.2 ± 10.3 vs. 20.7 ± 9.1, *p* = 0.042), with only six patients presenting mild side effects, i.e., dizziness, insomnia, fatigue, skin changes, constipation and hair loss. The retrospective, observational, chart by Ozudogru et al., 2020 [40] investigated the following three outcomes: 1. number of headache days; 2. number of weeks until wear-off of the benefit; 3. number of headache days after that the benefit wore off. According to the study results, there is potential for anti-CGRP mAbs to extend the therapeutic benefit of onabotulinumtoxinA and to delay the wear-off by average two weeks, when used in combination. The case series conducted by Silvestro et al., 2021 [50] displayed that the combination therapy could afford a significant reduction in MHDs (*p* < 0.01), in the intensity of headache during attacks (*p* < 0.01), in the need for symptomatic drugs per month (*p* < 0.01) and in migraine-induced disability according to MIDAS assessment (*p* < 0.01), with respect to the baseline and also to onabotulinumtoxinA or erenumab alone (*p* < 0.01). A total of 30% of patients reported pain in the injection sites in absence of serious adverse events. Finally, the study carried out by Toni et al. [51] reported a mean increase ranging from 3.8 to 12.6 headache-free days, depending on the antibody used in the combination therapy, among which fremanezumab was the most effective, without severe side effect, with the most common being constipation and injection site reactions. In all the studies the most commonly prescribed anti-CGRP mAb was erenumab, apart from what was observed in the study by Toni et al., 2021 [51], in which the most prescribed mAb was fremanezumab.

### 2.4. Quantitative Analysis

The present pooled analysis includes a sample of 665 patients from the eight studies included in the analysis. The results of the different primary and secondary outcomes, where comparable, were pooled. In particular, the 63.2-67% of patients treated with the combination therapy of anti-CGRP mAb and onabotulinumtoxinA experienced an improvement in mean MHDs at 30 days (Table 2). Moreover, a reduction in MMDs at 30 days was reported by 66.7–73.6% of patients (Table 2).

After 60 days of combined treatment it was possible to notice a change of 7.6 ± 8.3 in MHDs afforded to 81.1% patients by the administration of erenumab 70 mg in combination with onabotulinumtoxinA, instead of 7.2 ± 8.6 for 83.3% with onabotulinumtoxinA alone (Table 3); and a change of 6.8 ± 7.9 MMDs in 90.2% of treated patients with combination of erenumab 140 mg with onabotulinumtoxinA, in comparison with 6.7 ± 7.3 for 84.6% patients receiving onabotulinumtoxinA alone (Table 3).

The effect of improvement provided by the combination therapy of onabotulinumtoxinA with erenumab 70 mg on MHDs and of erenumab 140 mg on MMDs is confirmed at 90 days in 56.85% (instead of 43.6% of baseline) patients and in 34.1% (in comparison with 42.3% of baseline) patients, respectively (Table 4).

The combined treatment and follow-up up to 3–6–9–12 months afforded ≥50% monthly headache days/frequency reduction with respect to baseline in up to 58.8% of patients, with a pooled percentage of 35.5% after 6 months (Table 5).

The outcome of improvement of migraine-related disability was investigated through the assessment of a ≥30% improvement of MIDAS score in the studies by Blumenfeld et al., 2021 [47] and by Mechtler et al., 2022 [48]. However, in the study of Mechtler et al., MIDAS scores from baseline and post-index assessments were retrieved for only up to four patients; therefore, they were not reported and the available data come from the retrospective, longitudinal, chart study performed by Blumenfeld et al. A precentage of patients ranging from 27.1% to 31.0% achieved a ≥30% improvement of MIDAS score over baseline after 3 to 12 months of combined treatment (Table 6).

Data summarized in Table 7 refer to 6 months after combination therapy, since in the study by Mechtler et al. [48] a comparison of adverse effects with the study by Blumenfeld et al. [47] is reported, highlighting that the percentage of patients reporting adverse effects is considered at 6 months. The pooled data reveal a percentage of 13.3% patients experiencing adverse events that are not to be considered severe (Table 7).

### 2.5. Meta-Analysis

**Figure 3:**
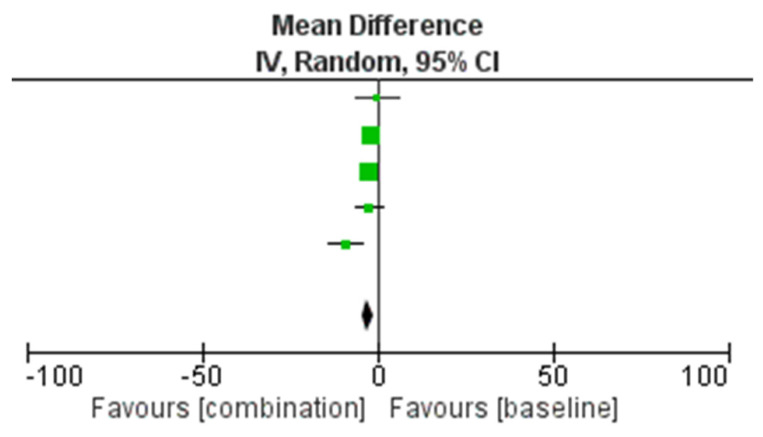
Forest plot of the results of the meta-analysis for the comparison of the onabotulinumtoxinA used in combination with anti-CGRP mAbs and alone about the efficacy primary outcome change in mean of monthly headache days (MHDs) after 3 months.

The most homogeneous outcome across the studies to conduct the meta-analysis is represented by the change in mean ± SD of MHDs after 3 months of combination treatment with onabotulinumtoxinA and anti-CGRP mAbs in comparison with baseline, consisting of the administration of the onabotulinumtoxinA alone. Therefore, the studies included in the meta-analysis are the following five out of the eight total studies: Armanious et al., 2021 [46], Blumenfeld et al., 2021 [47], Mechtler et al., 2022 [48], Nandyala et al., 2022 [49] and Toni et al., 2021 [51]. In particular, the study performed by Toni et al. [51] was included because the outcome was evaluated over 1–6 months of combined treatment, thus including 3-month assessment. The meta-analysis of mean outcome measures is reported in Table 8 and its forest plot is illustrated in Figure 3.

**Figure 4:**
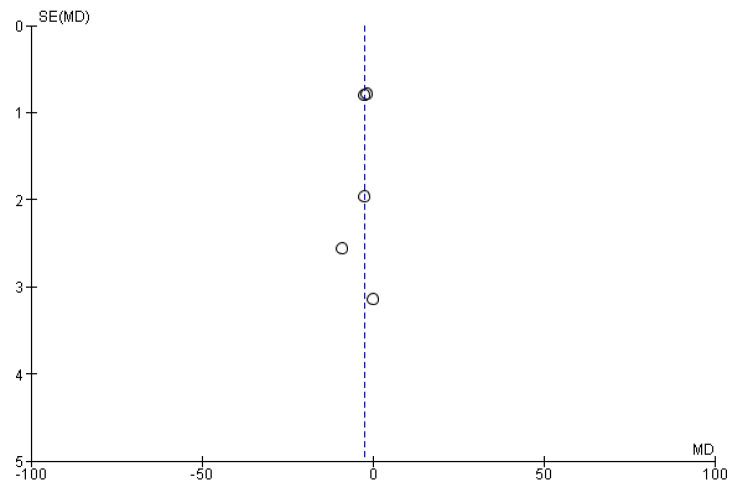
Funnel plot for publication bias assessment. The lack of asymmetry is suggestive of absence of publication bias. MD = mean difference; SE = standard error.

The results obtained in 393 total patients demonstrate the efficacy of the combined therapy instead of the treatment with onabotulinumtoxinA alone, in a statistically significant manner (*p =* 0.003) without significant heterogeneity (I^2^ = 49%). The width of the CIs and of the diamond shape support the reliability of the results. The funnel plot does not suggest publication bias (Figure 4).

### 2.6. Assessment of Certainty of Evidence

#### 2.6.1. Risk of Bias

**Figure 5:**
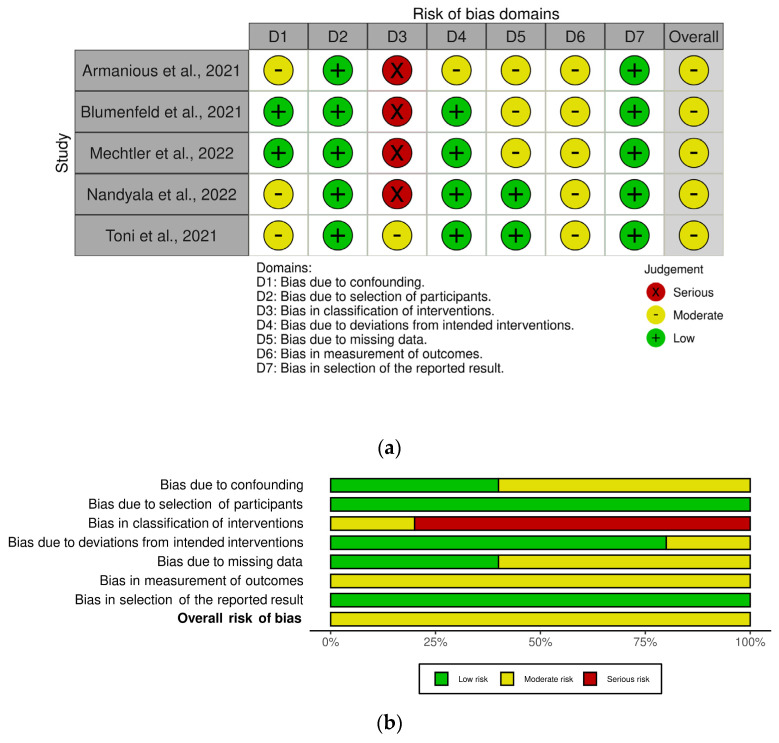
(**a**) Traffic-light plot and (**b**) summary plot of the risk of bias assessment of the studies included in the meta-analysis through ROBINS-I and graphed with robvis tool.

The risk of bias of the studies eligible for the present meta-analysis was assessed following the Risk Of Bias In Non-randomised Studies of Interventions (ROBINS-I) tool for the evaluation of effectiveness or safety (benefit or harm) of an intervention from nonrandomized studies of the effects of interventions (NRSI), e.g., observational studies including cohort studies and case-control studies, etc., typical of real-world evidence analysis. Seven domains were assessed: confounding and selection of participants (pre-intervention bias, differing from randomized trial bias assessment); classification of the interventions (at intervention bias, differing from randomized trial bias assessment); deviations from intended interventions, missing data, measurement of outcomes and selection of the reported result (postintervention bias, not differing from randomized trial bias assessment). The latter outcomes were rated as follows: 1. Confounding bias: factors that predict the outcome of interest also predict the intervention received at baseline; 2. Selection of participants bias: exclusion of some eligible participants or the initial follow-up time of some participants, as occurring when including already users rather than new users; 3. Bias in classification of interventions: misclassification of intervention status; 4. Bias due to deviations from intended interventions: systematic differences between groups in terms of care provided, representing a deviation from the intended intervention; 5. Missing data: loss to follow-up or exclusion of individuals with missing information; 6. Bias in measurement outcomes: outcome assessors aware of intervention status, different methods to assess outcomes in different groups or measurement errors; 7. Selective reporting of results. The rating followed four levels of judgement: low, moderate, severe and critical. Studies judged to be at low risk of bias for each domain/overall were comparable to a well-performed randomized trial with regard to the latter domain/overall, while studies deemed at critical risk of bias in at least one domain could not be included in the synthesis for that domain or at all in case of overall critical bias. The answers to the signaling questions were: “Yes, Y”; “Probably yes, PY”; “Probably no, PN”; “No, N”; and “No information, NI”. Lack of clear information in one or more key domains caused impossibility to rate the domain risk of bias. In fact, in all the studies with a longitudinal approach, patients allocated to the intervention group originated from a wider baseline group, though the sample size was small in the studies of Armanious et al., Nandyala et al., and Toni et al.Furthermore, the second domain is rated at “low risk of bias” since the occurring need for rescue medications different for type and quantity refers to issues of indirectness, assessed in the GRADE evaluation, and do not represent biases internal to the study, e.g., 61.5% of patients were actively using three or more other prophylactic migraine medications in the study by Armanious et al. Recall and information bias occur in the studies by Armanious et al., Blumenfeld et al., Mechtler et al. and Nadyala et al. Differential misclassification is present in all the studies, since none of the latter reports the absence of knowledge of the outcomes at the moment of the allocation to the intervention group. For the fourth domain, the study conducted by Armanious et al. is rated at “moderate risk of bias” since it is the only study among those included in the meta-analysis not to report de-identification of data. Loss to follow-up and exclusion of individuals with missing information occurred in the studies by Armanious et al., Blumenfeld et al. and Mechtler et al., arising attrition bias. Information about bias in measurement outcomes is lacking. Selective reporting never occurred. Therefore, the highest bias detected is pre-intervention bias due to the missing information linked to the retrospective design of the studies. The risk of bias assessment is illustrated in Figure 5.

#### 2.6.2. Summary of Findings (SoF) Grading of Recommendations Assessment, Development and Evaluation (GRADE)

**Figure 6:**
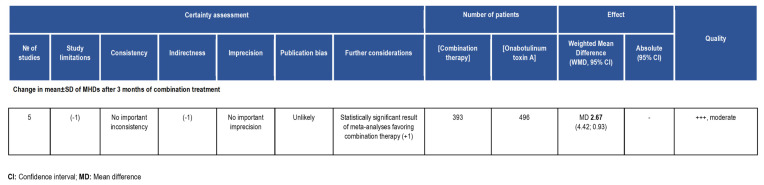
Summary of findings (SoF) illustrating the Grading of Recommendations Assessment, Development and Evaluation (GRADE) appraisal of moderate certainty of the body of evidence in favor of the treatment of chronic migraine with the combination therapy of onabotulinumtoxinA + anti-CGRP mAbs vs. onabotulinumtoxinA alone.

The study limitations domain downgrades all the studies included in the meta-analysis since they are observational and not randomized. In particular, the study by Armanious et al. is the only study that does not report de-identification of data and attrition bias occurred in the studies by Armanious et al., Blumenfeld et al. and Mechtler et al. However, the meta-analyses favor the intervention (combination therapy) rather than the baseline, thus upgrading the certainty of evidence. Consistency of results across studies is verified since heterogeneity I^2^ ranges from 47–49%. Lack of a real control arm in all the studies and of a record of concurrent treatments and comorbid conditions hamper generalizability of results of the studies of Armanious et al., Blumenfeld et al. and Mechtler et al., inducing indirectness. The small sample size of the studies of Armanious et al., Nandyala et al. and Toni et al. did not widen the overall CI, as supported by forest plot, not inducing imprecision. Publication bias was not found according to the funnel plot. The GRADE SoF is reported in Figure 6.

## 3. Discussion

This is the first systematic review and pooled analysis that intends to assess the efficacy and safety of the onabotulinumtoxinA in combination with anti-CGRP mAbs. From an initial screening of the 329 records identified through database searching, only 8 studies met the inclusion criteria and only 5 could be subjected to meta-analysis and critical appraisal with GRADE evaluation. This is the first obvious red flag that this combined therapy is poorly investigated. According to the summary of findings of the meta-analysis (Table 9), the PICOS question is answered to the outcome of change in mean ± SD of MHDs after 3 months of combination treatment with evidence for efficacy of the intervention vs. the comparison of moderate quality.

**Figure 7:**
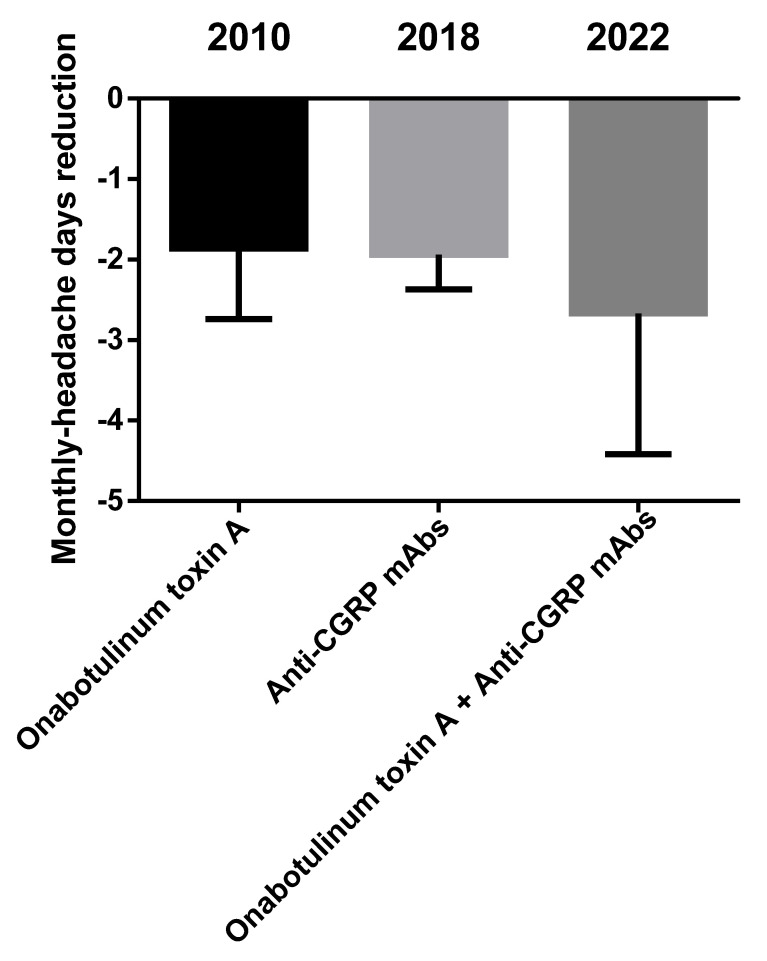
Benefit afforded by the combination therapy in comparison with the single treatments, onabotulinumtoxinA and anti-CGRP mAbs. Data are expressed as monthly headache days (MHDs) difference over baseline ± upper/lower limit of confidence interval (CI).

Within the eight retrieved studies, the outcome of the safety assessment revealed tolerability of the combined treatment with a pooled rate of ~13% patients developing adverse reactions that are not to be considered severe: the most common were constipation (often associated with the use of erenumab) and injection-site reactions. It is noticeable that outcome measures of migraine-induced disability, i.e., MIDAS and HIT-6 scores, were often lost to follow-up. In fact, the highest bias detected is pre-intervention bias due to the missing information linked to the retrospective design of the studies. The results are encouraging, since combined treatment proved to afford ≥50% monthly headache days/frequency reduction with respect to baseline in up to 58.8% of patients, with a pooled percentage of 35.5% after 6 months. Interestingly, in the study of Boudreau, the 65% of patients treated with the combination therapy obtained a reduction in migraine frequency, in comparison with erenumab affording efficacy only to the 26% of patients and erenumab in combination with prophylactic treatments other than botulinum toxin A to the 15%. Moreover, the retrospective observational chart by Ozudogru et al. highlighted the potential for anti-CGRP mAbs in combination with onabotulinumtoxinA to prolong its therapeutic benefit and to delay the wear-off by an average of two weeks. In contrast, the investigated Italian real-world setting did not report the presence of combined treatments of toxin with mAbs. The possible explanation for the effect of the combination of onabotulinumtoxinA and anti-CGRP mAbs, to exploit in resistant patients meeting disability criteria [52], could rely on a synergistic/additive effect of reversal of mechanical hypersensitivity of sensitized C-units and inhibition of the release of CGRP from meningeal and extracranial unmyelinated C-fibers by the onabotulinumtoxinA and of the action of the latter neuromodulator, as well as by means of receptor function blockade, by mAbs directed towards the ligand or the receptor. Furthermore, the neuronal/Schwann cell pathway can be involved in CGRP’s pro-nociceptive role [53]. In particular, fremanezumab, reported to be the most effective for the combined approach in the study by Toni et al., prevents the activation of Aδ- but not C-fibers, in contrast with the toxin that acts on C- but not Aδ-fibers [54]. Therefore, the present study answered the initial question to find out the MHD reduction afforded by the combination of the onabotulinumtoxinA with mAbs directed towards the signaling of CGRP. In fact, anti-CGRP mAbs were found to reduce the number of MHDs of 1.94 days from baseline and botulinum toxin of 1.86 days [36], and according to the results of this study, the combination therapy affords a reduction of 2.67 MHDs with respect to onabotulinumtoxinA alone (Figure 7).

Therefore, the present pooled analysis provides rational evidence for the need to rigorously test [52] the effectiveness and safety of the combination of the onabotulinumtoxinA with mAbs directed towards the signaling of CGRP to afford benefit to the significant proportion of patients not presenting clinical meaningful relief through available therapies. In particular, the study is supposed to be an adequately powered randomized, quadruple-masked, placebo-controlled, clinical trial assessing the rate of responders to the combination therapy with anti-CGRP mAbs and onabotulinumtoxinA presenting 30%, 50% and 75% reductions in MHDs and MMDs responder rates at 1–3 months with follow-up at 6, 9 and 12 months. A stratification analysis to allow comparison among the four different mAbs within the combination treatment should be planned. This clinical trial will provide definite data concerned with weighted mean difference (WMD) of MHDs and MMDs afforded by the combination therapy and to the rate of responders rescued within a resistant population who has not found relief yet to inform future medical decisions. Limitations of the present study rely in the retrospective, observational nature of the studies included causing missing information. Aged patients are often excluded from clinical trials [55], particularly on migraine for its rare occurrence in the over-50 population, although 85.9% of patients over 65 experience its onset just before 50 years of age, having medication-overuse headache (MOH) [56]. Thus, it is adviceable to include these patients often not receiving adequate pain control, mainly after stroke [57], in conditions of cognitive impairment [58,59,60] and since aging changes pain processing [61], and this issue is worsened during the pandemic [62,63].

### Registration and Protocol

The present systematic review and pooled analysis is registered and the protocol is available on the National Institute for Health Research (NIHR) International prospective register of systematic reviews PROSPERO with the number CRD42022313640.

## 4. Materials and Methods

### 4.1. Real-World Study Design

The real-world evidence was gathered through a retrospective study conducted in collaboration with the Calabrian pharmaceutic territorial service. Anonymized data were obtained through analytic search of the regional drug reimbursement and prescription repository for all the therapeutic plan prescriptions subjected to reimbursement by the National Health System (NHS). Based on EMA dosage indications, the medications searched over the period of 2020–2022 are onabotulinumtoxinA, erenumab (70 and 140 mg), galcanezumab (120 mg), fremanezumab (225 and 675 mg) and eptinezumab (100 mg). Since the latter drugs cannot be dispensed over the counter, the amount registered in this database corresponds unequivocally to the total of prescriptions to migraineurs suffering from over four migraine days per month, as per EMA indication. The health district includes a population of 298,000 inhabitants, of whom over 213,000 are under 60 years of age, i.e., the population most affected by migraine development. The need for written informed consent and ethical approval was waived owing to the retrospective use of anonymized data only. The study was conducted in accordance with the Declaration of Helsinki.

### 4.2. Objectives and Protocol

To the best of our knowledge, the present systematic review is the first aimed at verifying the working hypothesis that the concurrent therapy with onabotulinumtoxinA and monoclonal antibodies is effective and safe. This evidence could provide a synergic treatment option for patients difficult-to-treat and resistant to both classes of medications. The PRISMA recommendations [37,64,65] were followed to answer to the PICOS question. In particular, the intervention consists in anti-CGRP mAbs (fremanezumab, galcanezumab, eptinezumab and erenumab) administered in a combination protocol with onabotulinumtoxinA. Studies included comparing the intervention (anti-CGRP mAbs administered in a combination protocol with onabotulinumtoxinA) to placebo/no treatment or to an active control. Medications effective and approved for treatment and prevention of chronic migraine are considered active comparators. Studies eligible were clinical studies, prospective and retrospective. The efficacy primary outcome was a reduction in monthly headache days, a responder rate with 50% or a greater reduction in mean headache days per month; and the safety primary outcome was the absence of treatment-emergent adverse-events-related discontinuation and of serious or life-threatening adverse events. The reduction in pain severity and in headache duration and measures of disability, functioning and quality of life were the secondary outcomes. The protocol is registered in the International Prospective Register of Systematic Reviews, PROSPERO (CRD42022313640). The systematic review and pooled analysis were conducted in accordance to a protocol established prior to the literature search. In addition, the data extraction and selection process followed the PRISMA recommendations. Two members of the review committee independently screened titles and abstracts, followed by the full text of the studies, in agreement with the previously established inclusion and exclusion criteria. The reference lists of relevant papers were inspected for additional studies potentially missed in the database search. Any disagreement was solved by consensus or by consulting a third team member.

### 4.3. Inclusion Criteria

The analysis included patients suffering from chronic migraine, according to the International Headache Society (IHS, version 1-2-3-3b) criteria, of any age, ethnicity and gender, with clinical history of failure of previous treatments against migraine. No filters about study duration or follow-up and no restrictions concerned with publication date were applied. In vitro and in vivo animal studies, narrative or systematic reviews and meta-analysis, abstracts and congress communications, proceedings, editorials and book chapters, as well as studies not available in full text and not published in English were excluded from the analysis. The inclusion and exclusion criteria are illustrated in Table 10.

### 4.4. Information Sources

The systematic literature search screened PubMed/MEDLINE, Scopus, Web of Science, Cochrane Library databases (Cochrane Central Register of Controlled Trials-CENTRAL) for peer-reviewed studies published from the databases’ inception to present. A search for additional unpublished studies was conducted on the ClinicalTrials.gov registry. The search on databases was performed by two members of the review committee independently for records matching the search strings, from their inception to 19 April 2022, i.e., the date of last search.

### 4.5. Search Strategy

The following terms and modifications were used as search terms in combination for all the databases consulted, to be as extensive as possible aiming at high sensitivity/recall search strategy, keeping reasonable precision: “chronic migraine”, “onabotulinumtoxin (A)”, “botulinum toxin”, “anti-CGRP/(R) monoclonal antibodies”, “erenumab”, “galcanezumab”, “fremanezumab”, “eptinezumab”. Including both prospective and retrospective studies, no validated search filters for study design were applied. A different author (reviewer) from the two independently conducting the search (requestors), peer-reviewed that the search strategy could cover all the most relevant aspects, interpreting and addressing the research question appropriately, and the accuracy of lines and spelling of each search string, following the evidence-based guideline for Peer Review of Electronic Search Strategies (PRESS) for systematic reviews (SRs) [66].

### 4.6. Study Selection

The eligibility assessment of the studies was conducted independently by two authors to minimize the risk of excluding relevant records. Duplicate records were deleted through reference manager softwares (EndNote X7, Clarivate, London, UK) and title and abstract, and subsequently, the full texts were screened. The reference list of the articles was checked to extend and refine the search. There was overall consensus among all the authors, without the occurrence of relevant conflicts, previously planned to be solved through the Delphi method [67].

### 4.7. Data Synthesis, Risk of Bias Assessment and Critical Appraisal

The synthesis of the results was carried out in accordance with the Cochrane Consumers and Communication Review Group guidelines [68], considering the conversion of data into comparable measures and tabulating results of individual studies. In particular, data collected include: the report (author and year); the study design and sample size; the participants, based on type of migraine and of treatments for depression, and history of coronary artery disease; the research design with sampling, treatment assignment, allocation and concealment mechanisms, length of follow-up; the intervention type, timing and dose. The risk of bias (RoB) in the results of the individual studies and in the studies synthesis and the quality/certainty [69] of the body of evidence, according to PRISMA 2020 statement [42], were evaluated independently by two members of the review committee, based on the assessment of study limitations, missing or inadequate allocation concealment, absence of blinding, occurrence of selective outcome reporting bias, reduced sample for the effect or lack of sample size calculation. The revised Cochrane risk of bias tool RoB2 was planned to be used for randomized clinical trials (RCTs) [70], resulting in judgement (low; some concerns; high) for each specific outcome, according to the following items/domains: randomization process, deviations from intended interventions, missing outcome data, measurement of the outcome, selection of the reported result, overall risk of bias judgment summarizing across domains/components considering for each study the highest level of risk of bias reached in the domains. Any discrepancies in judgements of risk of bias were resolved by discussion for consensus between the two review authors, consulting a third author to solve any conflict, if necessary. For studies not belonging to the design of RCTs, specific methodological quality and risk of bias assessment tools for primary and secondary medical studies [71], e.g., the ROBINS-I tool [72], was used. The visualization of the risk of bias assement was conducted using the robvis visualization tool (Cochrane).

### 4.8. Statistical Analysis and Effect Measures

For the real-world pharmacoepidemiological data, the results extracted from the database were analyzed using Microsoft Office Excel 2010 (Microsoft, Milan, Italy) and evaluated statistically for differences using χ2 test for categorical variables, considering *p* < 0.05 significant, through GraphPad Prism^®^ 6.0 (GraphPad software Incorporated, San Diego, CA, USA). For the efficacy and tolerability outcome analyses, the number of events observed in a given treatment group across the studies was pooled and the results divided by the total number of patients included in the group [73], using SPSS-27 for Windows (IBM SPSS, Chicago, IL, USA). Due to the small number of studies meeting the inclusion criteria, and thus eligible for quantitative analysis, no sensitivity analysis (i.e., restricting the primary analysis to low-risk-of-bias studies) or following subgroup analysis or meta-regression based on stratification of the studies according to the judgement of the risk of bias were performed. Standardized mean differences and inverse variance were calculated for continuous variables through the Cochrane Review Manager 5.3 (RevMan5.3; Copenhagen: The Nordic Cochrane Center, The Cochrane Collaboration, Odense, Denmark). The heterogeneity of the studies was calculated through the random effect model [74] and the Higgins I^2^ value [75]. The publication bias was evaluated through the Egger’s linear regression test [76] for funnel plot asymmetry [77], adjusted through the “trim and fill” method [78]. The certainty of evidence of the selected outcomes was rated through the GRADE system [79], producing the SoF Table [80] through the evaluation of limitations, inconsistency, indirectness and publication bias using GRADE’s official software package GRADEpro GDT.

## Figures and Tables

**Table 1:** Characteristics of the eight studies meeting inclusion criteria for the analysis.

Study Report (Author and Year)	Study Design	Ethical Approval	Sample Size	Inclusion Criteria for Participants (Type of Migraine and of Treatments)	Intervention (*n*)	Control (*n*)	Intervention Type, Timing and Dose	Treatment Assignment, Allocation and Concealment Mechanisms	Outcome	Results	Length of Follow-Up	Limitations of the Study	Authors’ Conclusions
Armanious et al., 2021 [46]	Retrospective cross-sectional	Approved by the university’s Institutional Review Board, Pro00036880	All patients between the ages of 18 and 70 years of age seen in the university’s Headache Clinic (*n* = 78) with clinic encounters between 05/17/18 and 10/17/ 18. No sample power calculation	Patients between the ages of 18 and 70 years, with diagnosis of chronic migraine, defined as 15 or more headache days per month for three months with features of migraine headache on at least 8 days per month, and a baseline treatment with onabotulinumtoxinA for at least a nine-month duration. A total of 61.5% were actively using three or more other prophylactic migraine medications	*n* = 78	No placebo group for comparison. Comparator is represented by the patient’s baseline on onabotulinumtoxinA for a minimum of nine preceding months	Erenumab 70 mg (*n* = 37) and Erenumab 140 mg (*n* = 41) in addition to onabotulinumtoxinA injections. Time points = 30, 60 and 90 days	______	Primary outcome measure was monthly headache days (MHDs) and monthly migraine days (MMDs) at baseline, 30-, 60- and 90-days. MHDs and MMDs	Mean of 8.1 fewer MHDs (*p* < 0.001) and of 7.4 fewer MMDs (*p* < 0.001) at 90 days. Statistically significant 30% reduction at 90-days for migraine (*p =* 0.008), but not for headache; no statistically significant 50% reduction at 90-days for migraine or headache	Ninety days	Observational nature; lack of comparison group; lack of control of concurrent use of additional prophylactic migraine therapies; lack of control for co-morbid conditions; missing assessment of additional variables. Data were not analyzed for parameters with ≥50% missing data points	Erenumab in combination with onabotulinumtoxinA may enhance the effect on CGRP release from peripheral unmyelinated C fibers, blocking CGRP receptors in myelinated A-delta fibers. Clinically meaningful improvement in this intractable chronic migraineurs
Blumenfeld et al., 2021 [47]	Retrospective, longitudinal chart review	The study was conducted in accordance with International Council for Harmonisation guidelines and local legal requirements, and complied with the ethical principles of the World Medical Assembly. The New England Independent Review Board approved the study protocol and case report form (CRF) before study initiation	Patients aged ≥18 years referred at the Neurology Center of Southern California for chronic migraine (San Diego County, CA) between 1 October 2018, and 1 November 2019. No sample power calculation. A convenience sample of approximately 300 patients based on available charts and adequate sample size to characterize the safety profile was used	Adult patients (aged ≥ 18 years) with chronic migraine presenting at least two consecutive onabotulinumtoxinA treatment cycles without concomitant CGRP mAb therapy during the 8-month qualification period prior to the index date (the initiation of combination onabotulinumtoxinA and CGRP mAb therapy), and ≥1 month of subsequent combination treatment with onabotulinumtoxinA and CGRP mAb	*n* = 257	No placebo group for comparison. Comparator is represented by the patient’s baseline	Combination treatment of onabotulinumtoxinA with anti-CGRP mAbs (erenumab 70 (*n* = 136)/140 (*n* = 62) mg and galcanezumab 240 (*n* = 42) mg once monthly and fremanezumab 225 (*n* = 8)/675 (*n* = 7) mg once every three months as per label, instead of onabotulinum toxin not always administered per label, in dose ranging 115–200 U instead of 165U of baseline)	De-identified extracts of charts were prepared by site staff for the study	Monthly headache frequency, with intensity measured on a 0–10 scale. Migraine-related disability was captured on the Migraine Disability Assessment (MIDAS) questionnaire. Adverse events, discontinuations and reasons for discontinuation were recorded for each visit	Statistically significant and clinically meaningful reductions in mean MHDs at all visits. one-third (31.5–36.7%) of patients had a ≥50% reduction in MHDs after approximately 6 to 12 months: 43.7–45.1% of patients had a ≥5-point reduction from baseline, and 27.1–29.6% had a ≥30% reduction in MIDAS score. The mean MIDAS scores significantly decreased from baseline by 6.1 to 11.1 points during approximately 6 to 12 months of combination treatment. The 27.8% (68/245) of patients reported adverse events, with the most common being constipation (8.6% (21/245)), occurring most frequently in patients treated with erenumab (18/21). Concomitant use of other medications was recorded in 92.2% of patients at baseline, most commonly sumatriptan (20.7%) and topiramate (6.8%)	Twelve months	The onabotulinumtoxinA treatment was not always administered per label. The dates of migraine diagnosis, initiation of onabotulinumtoxinA, and headache frequency prior to onabotulinumtoxinA treatment prior to the 8-month qualification period were collected aas available. Missing data due to loss to follow-up were not included	The real-world data demonstrated that combination use of onabotulinumtoxinA and a CGRP mAb was generally well tolerated and suggestive of additive or synergistic benefit in headache frequency and migraine-related disability
Boudreau 2020 [41]	Prospective, observational study (NCT04152434)	All patients consented to participate to the study	No sample power calculation	Chronic migraineurs with migraine 15–30 days per month at baseline with or without an actual preventive drug, who failed more than 3 preventive drugs previously, naïve to monoclonal anti-CGRP mAbs	*n* = 69 nonpresenting reduction in migraine frequency at baseline out of *n* = 158 nonresponders	Group I On no preventive therapy at the start of Erenumab, (no Botox cohort) Group II On Botulinum Toxin type A prior to the add on therapy with Erenumab (Botox cohort). Group III On an oral preventive therapy prior to the add on therapy with Erenumab (no Botox cohort)	Botulinum Toxin type A + erenumab (70/140 mg)	______	The primary objective, was the reduction in the frequency of monthly migraine days. Adverse events were a secondary outcome	Forty-five patients (65%) experienced a decrease in the frequency of their monthly migraine days by 5–7 days, becoming episodic. Seventy-two adverse events were experienced during the 9 months of treatment, 56 events with the 140 mg. dose (118 patients), and 16 events with the 70 mg. dose (40 patients), the most frequent being comnstipation (34% of patients)	Nine months	Fifteen patients were lost to follow up. Fifty seven percent of patients failed to reach the primary end point	The 65% of patients receiving combination therapy achieved reduction in migraine frequency, instead of the 26% with erenumab alone or the 15% with erenumab in combination with prophylactic treatments other than botulinum toxin A
Mechtler et al., 2022 [48]	Retrospective, noninterventional, longitudinal study	The New England Independent Review Board (IRB) reviewed the study protocol prior to study initiation and determined the study as exempt from review. This study was conducted in accordance with current applicable regulations, International Conference of Harmonization guidelines, and local legal requirements, and complies with the ethical principles of the World Medical Assembly	All the eligible patients treated at the DENT Headache Center (Buffalo, NY, USA) between 1 June 2018 and 15 March 2020. The index date was defined as the start of combination treatment with onabotulinumtoxinA and a CGRP mAb and occurred between 1 June 2018 and 15 March 2019. The target sample size was up to ~300 patients, the expected number of eligible patients at the site	Adult patients (≥18 years) with chronic migraine treated with ≥2 consecutive cycles of onabotulinumtoxinA before ≥1 month of continuous onabotulinumtoxinA and CGRP mAb (erenumab, fremanezumab, or galcanezumab) combination treatment	*n* = 148	No placebo group for comparison. Comparator is represented by the patient’s baseline. A baseline period of 1–3 months prior to index was used to assess the effectiveness of onabotulinumtoxinA treatment monotherapy. At baseline most used concomitant migraine medications (*n* = 143/148, 96.6%) and presented comorbid conditions (*n* = 142/148, 95.9%)	Continuous onabotulinumtoxinA and CGRP mAb [erenumab (70–140 mg), fremanezumab (225 mg), or galcanezumab (120 mg)] combination treatment	De-identified data were used	Headache frequency (monthly headache days). The effect on quality of life and disability was assessed with the 6-Item Headache Impact Test (HIT-6) and Migraine Disability Assessment (MIDAS), respectively. Adverse and serious adverse events were reported	After 12 months of combination therapy, MHD decreased by a mean of 4.6 days (95% CI 2.5–6.7). The 34.9% (95% CI 21.0–50.9) patientsachieved ≥50% reduction in MHD. Adverse events were reported by 18 patients (12.2%), with the most common being constipation (*n* = 8, 5.4% [onabotulinumtoxinA plus erenumab only]) and injection site reactions (*n* = 5, 3.4%)	Twelve months	Per label, erenumab, fremanezumab, and galcanezumab were administered once monthly, while OnabotulinumtoxinA was not always administered per label. Results were based on available data and missing data were not included. In fact, since paired HIT-6 and MIDAS scores from baseline and post-index assessments were only available for up to four patients, no further analyses were reported for those outcome measures	Incremental and clinically meaningful reductions in MHD are provided by combination therapy
Nandyala et al., 2022 [49]	Retrospective, cohort study	The study was approved by Institutional Review Board, and patient consent was deemed not needed. However, before the beginning of the therapy with erenumab, patients were provided information on expected side effects	Patients at Medstar Georgetown Headache Center. No sample size calculation	Adult (≥18 years old) patients who had a diagnosis of chronic migraine receiving onabotulinumtoxinA	*n* = 50 (2 patients started with 70 mg erenumab and moved to the 140 mg group)	No placebo group for comparison. Comparator is represented by the treatment with onabotulinumtoxinAlone	Erenumab [70 (*n* = 22)/140 (*n* = 26) mg) in combination with onabotulinumtoxinA, *n* = 50	All data were de-identified, collected and recorded in a password protected document	Primary endpoint was decrease in number of migraine days. Secondary endpoints included a decrease in headache days and reported side effects	Significant reduction in MMDs (11.3 ± 9.3 vs. 14.9 ± 9.4, *p* < 0.001) and of MHDs (18.2 ± 10.3 vs. 20.7 ± 9.1, *p* = 0.042); 6 patients reported mild side effects including dizziness, insomnia, fatigue, skin changes, constipation and hair loss	One month	Data about demographic characteristics, other prophylactic medications, co-morbidities and number of prior treatments were not gathered	Erenumab and onabotulinumtoxinA, when used in combination, Show a decrease in migraine days per month and in headache days per month, without severe side effects
Ozudogru et al., 2020 [40]	Retrospective, observational, chart	_____	Patients diagnosed with chronic migraine, having received at least two onabotulinumtoxinA treatments, after June 2018, and currently prescribed erenumab, fremanezumab or galcanezumab. No sample power calculation	Patients with a diagnosis of chronic migraine, who received at least two onabotulinumtoxinA treatments, after June 2018, and currently prescribed erenumab, fremanezumab or galcanezumab	*n* = 36	No placebo group for comparison. Comparator is represented by the treatment with onabotulinumtoxinAlone	OnabotulinumtoxinA in combination with erenumab, fremanezumab or galcanezumab	______	1. number of headache days; 2. number of weeks until the benefit from wear-off; 3. number of headache days after the benefit wore off	Half of the patients (*n* = 18) demonstrated improvement in headache burden >50% after the addition of an anti-CGRP mAb and an average increase of 2.0 weeks taken to wear-off during combination treatment	______	Small sample size. Retrospective, single-site study. Answers to the pre-procedure questionnaire used were based on the patients’ own recollection of events, with potential for recall bias	Potential for anti-CGRP mAbs to prolong the therapeutic benefit of onabotulinumtoxinA and to delay the wear-off by average two weeks
Silvestro et al., 2021 [50]	Case series	Approved by Ethical Committee of the University of Campania Luigi Vanvitelli. Each patient gave informed consent	No sample power calculation	Patients, aged between 18 and 65 years, who failed at least four or more oral preventive medication classes (propranolol or metoprolol, topiramate, flunarizine, valproate, amitriptyline, or candesartan) due to lack of efficacy or intolerable side effects, prescribed with onabotulinumtoxinA for at least 9 months (e.g., three administrations of 185 UI), interrupted in favor of a 6-month erenumab 140 mg monthly administration	*n* = 10	No placebo group for comparison. Comparator is represented by baseline	Combined treatment with onabotulinumtoxinA (185 UI quarterly administration) and erenumab (140 mg monthly administration)	______	MHDs, severity of headache during attacks, symptomatic drug intake per month, and migraine disability	Statistically significant reduction of MHDs (*p* < 0.01), intensity of headache during attacks (*p* < 0.01), and symptomatic drug intake per month (*p* < 0.01), as well as MIDAS-assessed migraine disability (*p* < 0.01), compared to the baseline and also to onabotulinumtoxinA or erenumab alone (*p* < 0.01). The 30% of patients reported pain in the injection sites, without serious adverse events	Six months	Small sample size	A combined therapy may provide an additive or synergistic effect on the trigeminal nociceptive pathway
Toni et al., 2021 [51]	Case series	No approval since the study is based on authors’ clinical experience	Patients admitted between May 2018 to June 2020. No sample power calculation	Chronic migraine with suboptimal response to onabotulinumtoxinA	*n* = 17	No placebo group for comparison. Comparator is represented by response to onabotulinumtoxinA alone	Combined therapy with onabotulinumtoxinA and fremanezumab (*n* = 9), erenumab (*n* = 4) or galcanezumab (*n* = 4)	Patients’ records confidentiality was maintained and data de-identified	Headache days and severity over 1–6 months	A mean improvement of +12.6 headache-free days was observed in fremanezumab patients, +6.4 in erenumab patients, and +3.8 in galcanezumab patients, for a total improvement experienced by *n* = 11 patients. No severe adverse side effects were experienced, with only mild irritation at the injection site and constipation. The response rate resulted of 58.82% for headache days reduction and of 64.71% for headache severity	Six months	Placebo-controlled, randomized studies are required to confirm the results	Patients suffering from severe, intractable migraine may benefit from onabotulinumtoxinA and anti- CGRP mAb dual therapy, likely due to a synergistic mechanism at receptor and ligand level

**Table 2:** Changes in monthly headache and migraine days (Mean ± SD of MHDs and MMDs) at 30 days.

Study Report (Author and Year)	Intervention Dose 1 Change in MHDs	Intervention Dose 2 Change in MHDs	Intervention Dose 1 Change in MMDs	Intervention Dose 2 Change in MMDs
Armanious et al., 2021 [46]	Erenumab 70 mg	Erenumab 140 mg	Erenumab 70 mg	Erenumab 140 mg
	*n* = 33/37	*n* = 39/41	*n* = 32/37	*n* = 41/41
	6.8 ± 7.5	6.8 ± 8.0	9.6 ± 9.4	7.5 ± 7.1
	Improvement in 89.2% of treated patients	Improvement in 95.1% of treated patients	Improvement in 86.5% of treated patients	Improvement in 100% of treated patients
Nandyala et al., 2022 [49]	*n* = 22/50 (same treatment)	*n* = 22/50 (same treatment)	*n* = 26/50 (same treatment)	*n* = 26/50 (same treatment)
	2.5 ± 1.2	2.5 ± 1.2	3.6 ± 0.1	3.6 ± 0.1
	Improvement in 44% of treated patients	Improvement in 44% of treated patients	Improvement in 52% of treated patients	Improvement in 52% of treated patients
Pooled results	Improvement in 63.2% of treated patients	Improvement in 67% of treated patients	Improvement in 66.7% of treated patients	Improvement in 73.6% of treated patients

**Table 3:** Changes in monthly headache and migraine days (Mean ± SD of MHDs and MMDs) at 60 days.

Study Report (Author and Year)	Comparator/Baseline Change in MHDs	Comparator/Baseline Change in MMDs	Intervention Dose 1 Change in MHDs	Intervention Dose 2 Change in MHDs	Intervention Dose 1 Change in MMDs	Intervention Dose 2 Change in MMDs
Armanious et al., 2021 [46]	*n* = 65/78 7.2 ± 8.6	*n* = 66/78 6.7 ± 7.3	Erenumab 70 mg *n* = 30/37 7.6 ± 8.3 Improvement over baseline in 81.1% of treated patients	Erenumab 140 mg *n* = 35/41 6.9 ± 9.0	Erenumab 70 mg *n* = 29/37 6.6 ± 6.5	Erenumab 140 mg *n* = 37/41 6.8 ± 7.9 Improvement over baseline in 90.2% of treated patients

**Table 4:** Changes in monthly headache and migraine days (Mean ± SD of MHDs and MMDs) at 90 days.

Study Report (Author and Year)	Comparator/Baseline Change in MHDs	Comparator/Baseline Change in MMDs	Intervention Dose 1 Change in MHDs	Intervention Dose 2 Change in MHDs	Intervention Dose 1 Change in MMDs	Intervention Dose 2 Change in MMDs
Armanious et al., 2021 [46]	*n* = 34/78 8.1 ± 8.8	*n* = 33/78 7.4 ± 6.8	Erenumab 70 mg *n* = 21/37 8.3 ± 8.7 Improvement over baseline in 56.8% of treated patients	Erenumab 140 mg *n* = 13/41 7.8 ± 9.3	Erenumab 70 mg *n* = 19/37 6.7 ± 5.6	Erenumab 140 mg *n* = 14/41 8.4 ± 8.2 Improvement over baseline in 34.1% of treated patients

**Table 5:** The percentage of patients presenting ≥50% monthly headache frequency reduction after 6 months of treatment reaches the 58.8% and pooled results across studies amounts to 35.5%.

Study Report (Author and Year)	3 Months	6 Months	9 Months	12 Months
Blumenfeld et al., 2021 [47]	25.7% (*n* = 56/218)	36.7% (*n* = 66/180)	33.3% (*n* = 47/141)	31.5% (*n* = 33/106)
Mechtler et al., 2022 [48]	21.2% (*n* = 24/113)	28.9% (*n* = 26/90)	29.0% (*n* = 20/69)	34.9% (*n* = 15/43)
Toni et al., 2021 [51]	____________	58.8% (*n* = 10/17)	____________	____________
Pooled results		35.5% (*n* = 102/287)		

**Table 6:** The highest percentage of patients presenting ≥30% improvement of MIDAS score is 31.0% after 3 months of combination therapy of onabotulinumtoxinA with anti-CGRP mAbs.

Study Report (Author and Year)	3 Months	6 Months	9 Months	12 Months
Blumenfeld et al., 2021 [47]	31.0% (*n* = 43/139)	29.6% (*n* = 33/112)	29.4% (*n* = 24/83)	27.1% (*n* = 18/66)
Mechtler et al., 2022 [48]	____________	____________	____________	____________

**Table 7:** Adverse events after 6 months of combined therapy of onabotulinumtoxinA and anti-CGRP mAbs occur in a percentage of patients ranging from 12.1% to 14.2%.

Study Report (Author and Year)	6 Months
Blumenfeld et al., 2021 [47]	14.2% (*n* = 28/197)
Mechtler et al., 2022 [48]	12.1% (*n* = 18/148)
Pooled results	13.3% (*n* = 46/345)

**Table 8:** Meta-analysis of the data from the five studies included in the quantitative analysis for the efficacy primary outcome change in mean of monthly headache days (MHDs) after 3 months of combination treatment with onabotulinumtoxinA and anti-CGRP mAbs.

	OnabotulinumtoxinA + mAb			OnabotulinumtoxinA				Mean Difference
Study or Subgroup	Mean	SD	Total	Mean	SD	Total	Weight	IV, Random, 95% CI
Armanious et al., 2021	14.2	11.5	21	14.2	11.1	35	6.9%	0.00 [−6.14, 6.14]
Blumenfeld et al., 2021	10.3	8	180	12.1	8	246	34.8%	−1.80 [−3.34, −0.26]
Mechtler et al., 2022	11.6	6.3	127	14	6.9	148	34.5%	−2.40 [−3.96, −0.84]
Nandyala et al., 2022	18.2	10.3	48	20.7	9.1	50	14.3%	−2.50 [−6.35, 1.35]
Toni et al., 2021	18.6	9.4	17	27.6	4.8	17	9.6%	−9.00 [−14.02, −3.98]
**Total (95% CI)**			**393**			**496**	**100.0%**	**−2.67 [−4.42, −0.93]**

Heterogeneity: Tau^2^ = 1.54; Chi^2^ = 7.81, df = 4 (*p =* 0.10); I^2^ = 49%; Test for overall effect: Z = 3.01 (*p =* 0.003).

**Table 9:** Summary of the aim of the study and of the quantitative findings.

[Combination therapy of onabotulinumtoxinA + anti-CGRP mAbs] compared with [baseline] for [chronic migraine]		
**Patient or population:** [patients] with [chronic migraine] **Settings:** [real-world] **Intervention:** [**Combination therapy of onabotulinumtoxinA + anti-CGRP mAbs**] **Comparison:** [**OnabotulinumtoxinA alone**]		
**Outcomes**	**Effect (95% CI)**	**Quality of the evidence (GRADE)**
**Change in mean ± SD of MHDs after 3 months of combination treatment**	MD −2.67, 95% CI −4.42 to −0.93; participants = 393 intervention and 496 baseline; studies = 5; I^2^ = 49%	⊕⊕⊕⊝ **moderate**
**CI:** Confidence interval; **MD:** Mean difference.		
GRADE Working Group grades of evidence **High quality:** Further research is very unlikely to change our confidence in the estimate of effect. **Moderate quality:** Further research is likely to have an important impact on our confidence in the estimate of effect and may change the estimate. **Low quality:** Further research is very likely to have an important impact on our confidence in the estimate of effect and is likely to change the estimate. **Very low quality:** We are very uncertain about the estimate.		

**Table 10:** Inclusion and exclusion criteria.

Inclusion Criteria	Exclusion Criteria
Patients suffering from chronic migraine, according to the International Headache Society (IHS, version 1-2-3-3b) criteria, of any age, ethnicity and gender; Clinical history of failure of previous treatments against migraine; No filters about study duration or follow-up; No restrictions concerned with publication date.	In vitro and in vivo animal studies, narrative or systematic reviews and meta-analysis, abstracts and congress communications, proceedings, editorials and book chapters; Studies not available in full text; Studies not published in English.

## Data Availability

The data presented in this study are available within the article.
